# Three-dimensional reconstruction of industrial parts from a single image

**DOI:** 10.1186/s42492-024-00158-7

**Published:** 2024-03-27

**Authors:** Zhenxing Xu, Aizeng Wang, Fei Hou, Gang Zhao

**Affiliations:** 1 School of Mechanical Engineering and Automation, Beihang University, Beijing, 100191 China; 2 Key Laboratory of Aeronautics Smart Manufacturing, Beihang University, Beijing, 100191 China; 3 State Key Laboratory of Computer Science, Institute of Software, Chinese Academy of Sciences, Beijing, 100190 China

**Keywords:** Three-dimensional reconstruction, Non-uniform rational B-splines, Industrial parts, Deep learning

## Abstract

This study proposes an image-based three-dimensional (3D) vector reconstruction of industrial parts that can generate non-uniform rational B-splines (NURBS) surfaces with high fidelity and flexibility. The contributions of this study include three parts: first, a dataset of two-dimensional images is constructed for typical industrial parts, including hexagonal head bolts, cylindrical gears, shoulder rings, hexagonal nuts, and cylindrical roller bearings; second, a deep learning algorithm is developed for parameter extraction of 3D industrial parts, which can determine the final 3D parameters and pose information of the reconstructed model using two new nets, CAD-ClassNet and CAD-ReconNet; and finally, a 3D vector shape reconstruction of mechanical parts is presented to generate NURBS from the obtained shape parameters. The final reconstructed models show that the proposed approach is highly accurate, efficient, and practical.

## Introduction

With the development of intelligent manufacturing, mechanical product production has gradually become increasingly automated, flexible, intelligent, and highly integrated. Thus, artificial intelligence, including three-dimensional (3D) reconstruction and sample data acquisition, is inevitably used. For instance, when manipulators are used for automatic loading and unloading, it is necessary to obtain the 3D data of parts (3D reconstruction) from an image to grasp the object. For parts with irregular surfaces and features that are difficult to measure directly, it is necessary to perform a reverse reconstruction to obtain the size parameters. Current 3D reconstruction methods mostly obtain point cloud data through 3D scanning and thereafter achieve shape reconstruction by post-processing the point cloud. However, it is difficult to achieve real-time performance and vector reconstruction using this approach. This study proposes an image-based 3D vector reconstruction of typical mechanical products that is highly efficient and can achieve non-uniform rational B-splines (NURBS) based reconstruction with high fidelity, simplicity, and inexpensive consumer cameras.

**Fig. 1:**
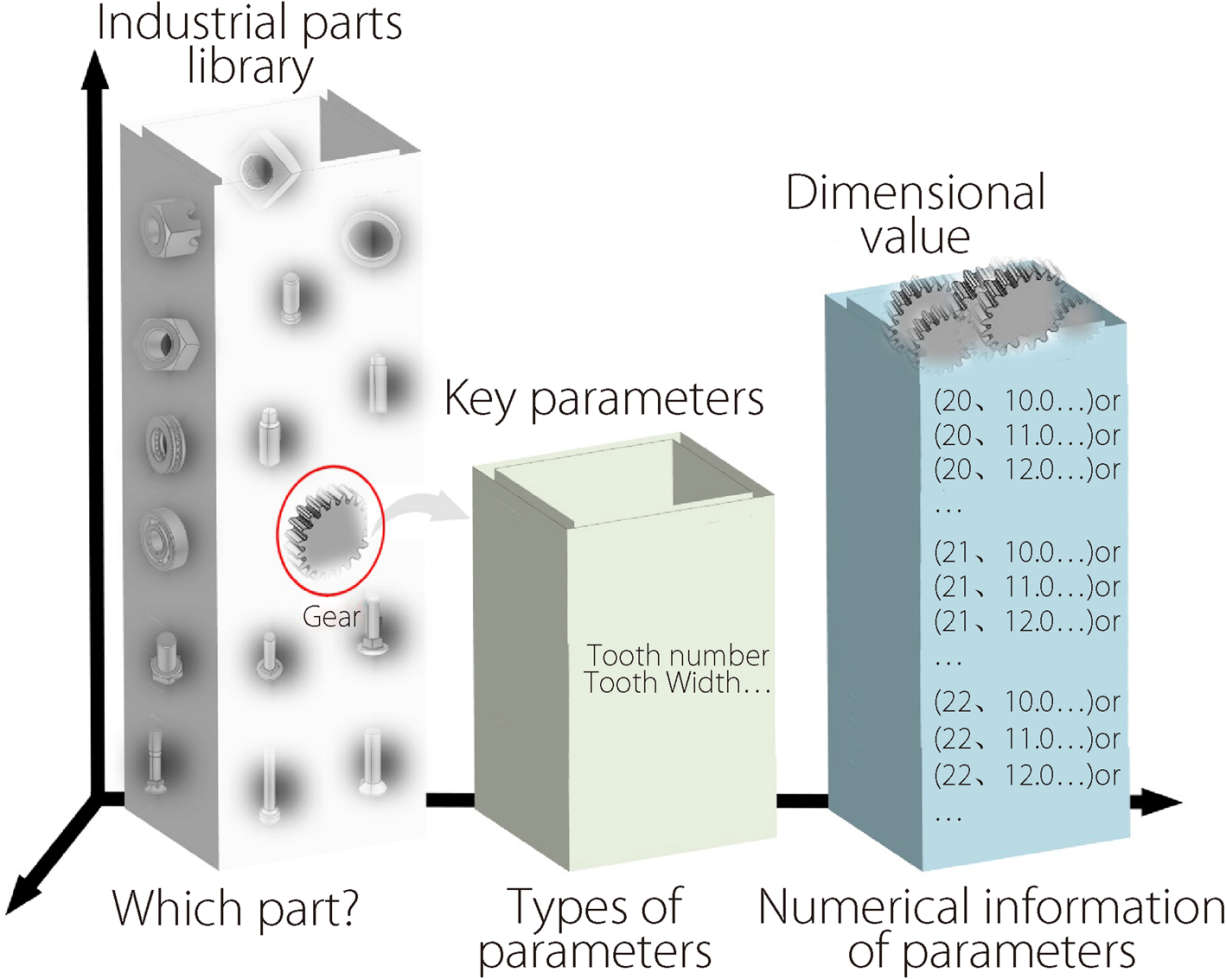
Parameters of different industrial parts

In the Standard for the Exchange of Product Model Data issued by the International Organization for Standardization, NUBRS is the only mathematical method used to define the geometric shapes of industrial products. In digital manufacturing [1], all industrial parts have a unified mathematical expression known as NUBRS [2]. In the design and manufacturing processes, NURBS is used not only for computer aided design (CAD) but also for data exchange. For industrial parts, the generation of CAD models depends on the corresponding parameters, such as tooth number, modulus, and tooth width of gears. In general, the parameters depend on the classification of the industrial parts, and the only difference is the value of each parameter (Fig. 1). Therefore, when the type of industrial part is determined, it is possible to reconstruct an accurate 3D model of the part based on NURBS in case its parameters are obtained.

In this study, the research interest was the 3D reconstruction of industrial parts. The contributions of this study are as follows: (1) A dataset of two-dimensional (2D) images of typical industrial parts is constructed, including hexagonal head bolts, cylindrical gears, shoulder rings, hexagon nuts, and cylindrical roller bearings. (2) A deep learning algorithm for parameter extraction of 3D industrial parts that can determine the final 3D parameters and pose information of the reconstructed model using two new nets is developed: a class prediction net (CAD-ClassNet) and a reconstruction prediction net (CAD-ReconNet). CAD-ClassNet was used to determine the type of reconstructed part, and the part parameters were predicted using CAD-ReconNet. (3) A NURBS-based 3D reconstruction of the parts from the parameters obtained by deep learning is presented.

3D reconstruction is classical in computer vision and is widely used in the fields of automatic driving and intelligent robots. Current 3D reconstruction methods based on 2D images can be classified into traditional multiple view geometry approaches [3–5] and deep learning-based methods [6–17]. The former primarily uses a stereo-matching algorithm to recover the 3D structure from a series of 2D images from multiple views obtained by a camera. However, they cannot recover 3D shapes from a single view. Deep learning-based methods could encode prior knowledge into the network such that they are able to reconstruct the 3D model from a single image. Since AlexNet was first proposed [18], the architecture of deep learning networks has been continuously developing [19–23]. Deep learning has a strong learning ability and good portability, making it easy to achieve excellent results in image classification [20, 21], target detection [19], and image denoising [22]. Deep learning-based single-view 3D stereo methods exhibit better performance than traditional approaches.

Researchers have reconstructed 3D models based mainly on the 2D information fusion of two or multiple views. Jia et al. [7] proposed a dual-view network, DV-NET, which fuses point clouds with two different views using a point-cloud fusion network. Soltani et al. [12] trained data using a depth map and a contour map of multiple views and generated 3D shapes with more details to achieve high-fidelity modeling. Multiple-view-based 3D reconstruction methods have achieved better results; however, it is more challenging to reconstruct 3D shapes from a single image. Single-view-based 3D reconstruction has been applied to buildings [13], furniture [15], human bodies [16], porous media [17], and other structures, particularly indoor furniture. However, they are not applicable to vector model reconstruction, particularly in intelligent manufacturing and mechanical areas.

The learning ability of a deep learning network relies mainly on a large amount of data. Current 3D reconstruction methods use ShapeNet [24], ObjectNet3D [25], and Pix3D [26] for training. For the aforementioned datasets, the 2D images were aligned with the 3D model using marked points, and different alignment methods were used to improve the reconstruction accuracy. However, it is difficult to fundamentally remove alignment deviations using these methods. Based on MarrNet [11], Sun et al. [26] proposed an approach for shape reconstruction and pose estimation using a 2D–3D alignment dataset. However, these reconstruction methods rely on highly accurate datasets, and it is difficult or expensive to obtain related sample data. Moreover, for shape reconstruction, voxels [27], point clouds [28], and grids [29] have been used to represent reconstructed 3D objects. Although these representations transmit 3D models in a neural network, the final results are not sufficiently accurate without semantic information, and the computation is expensive. Based on NURBS, a unique mathematical representation of industrial products, this study obtained a 3D vector reconstruction of typical industrial parts from a single image. The proposed approach was more efficient, and the final results achieved high accuracy.

## Methods

### Industrial part dataset generation

For deep learning, its excellent ‘learning’ ability is mostly owing to the training of a large number of samples [30–32]. In practical applications, industry-related historical data can be used to construct training datasets. However, additional industrial sample data are difficult to obtain because of sample acquisition and statistics in additional industries, which hardly meet the large amount of training data required for deep learning. Therefore, obtaining sample sets is closely related to the application of deep learning in industry.

In this study, a 2D image dataset of industrial parts of different sizes and views was constructed, which can be used to construct a feature library of reconstruction parameters. However, it is tedious to obtain a 2D dataset by actual photography, and the accuracy of the camera influences the quality of the sample data, or even the efficiency and accuracy of the final 3D reconstruction. Considering the limitations of actual photography, a CAD model omnidirectional photography approach that can automatically obtain an image dataset of industrial parts with different sizes and poses is proposed in this study.

The size of the input images influences the accuracy of 3D reconstruction and the feasibility of data training in deep learning. The sample sizes are as follows: In this study, the basic idea of 3D reconstruction was to extract the features of sample images through the employed neural network, and thereafter obtain the 3D parameters through a new algorithm by feature analysis. The relationships between these parameters are as follows:

1 $$n = s/\left( {0.5 \times a} \right)$$

where *s* denotes the size of the parts, 0.5 is the proportion of the model in the image, *a* denotes the accuracy of the parameters, and *n* denotes the resolution of the image.

The high resolution of sample images will lead to ‘explosion’ of the equipment in data training, and it is difficult for the network to train and fit. Herein, the accuracy of the 3D reconstruction is set to 0.1 mm, and the analysis accuracy of the network is expected to be at the pixel level; thus, the resolution of the input image was 200 × 200 by Eq. (1) when the model size was 10 mm. The resolution of the input image was 256 × 256 pixels. Therefore, the size of the original images was set to 256 × 256 pixels. Additionally, a method to automatically implement the omnidirectional photography of industrial models and successfully obtain a large number of 2D images with a white background is presented. Thereafter, a construction approach for image datasets of industrial parts that is adequate for deep learning in industrial fields is proposed.

**Fig. 2:**
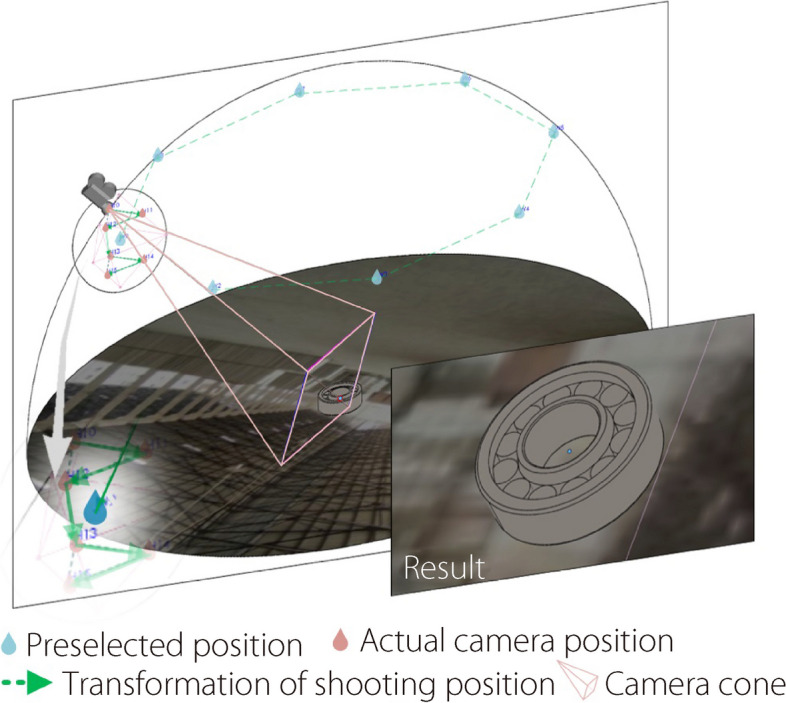
Virtual photography of industrial parts

To illustrate this further, five typical industrial parts (Table 1) were selected, and each type had ten sizes and 336 poses. Finally, the number of 2D images of the industrial parts dataset was 336 × 5 × 10 = 16,800, where the total shooting points were 7 × 8 × 6 = 336, associated with seven latitude lines, eight longitude lines, and six customary shooting points in the virtual photography space (Fig. 2).

**Table 1:** Five typical industrial parts

Industrial parts	Parameters	Parameter values
Hexagon head bolt	[length]	Type_1_ = [15.00] Type_*i*_ = Type_1_ + [1.00] × (*i - *1)
Cylindrical gear	[modulus; tooth width]	Type_1_ = [1.50, 7.50] Type_*i*_ = Type_1_ + [0.10, 0.50] × (*i* - 1)
Shoulder ring	[external diameter; thickness]	Type_1_ = [22.00, 3.25] Type_*i*_ = Type_1_ + [1.00, 0.15] × (*i* - 1)
Hexagon nut	[nominal diameter; height]	Type_1_ = [11.00, 8.40] Type_*i*_ = Type_1_ + [1.00, 0.76] × (*i* - 1)
Cylindrical roller bearing	[external diameter; thickness]	Type_1_ = [30.00, 10.00] Type_*i*_ = Type_1_ + [1.00, 0.33] × (*i* - 1)

where *i* = 1, 2, ……, 10

**Fig. 3:**
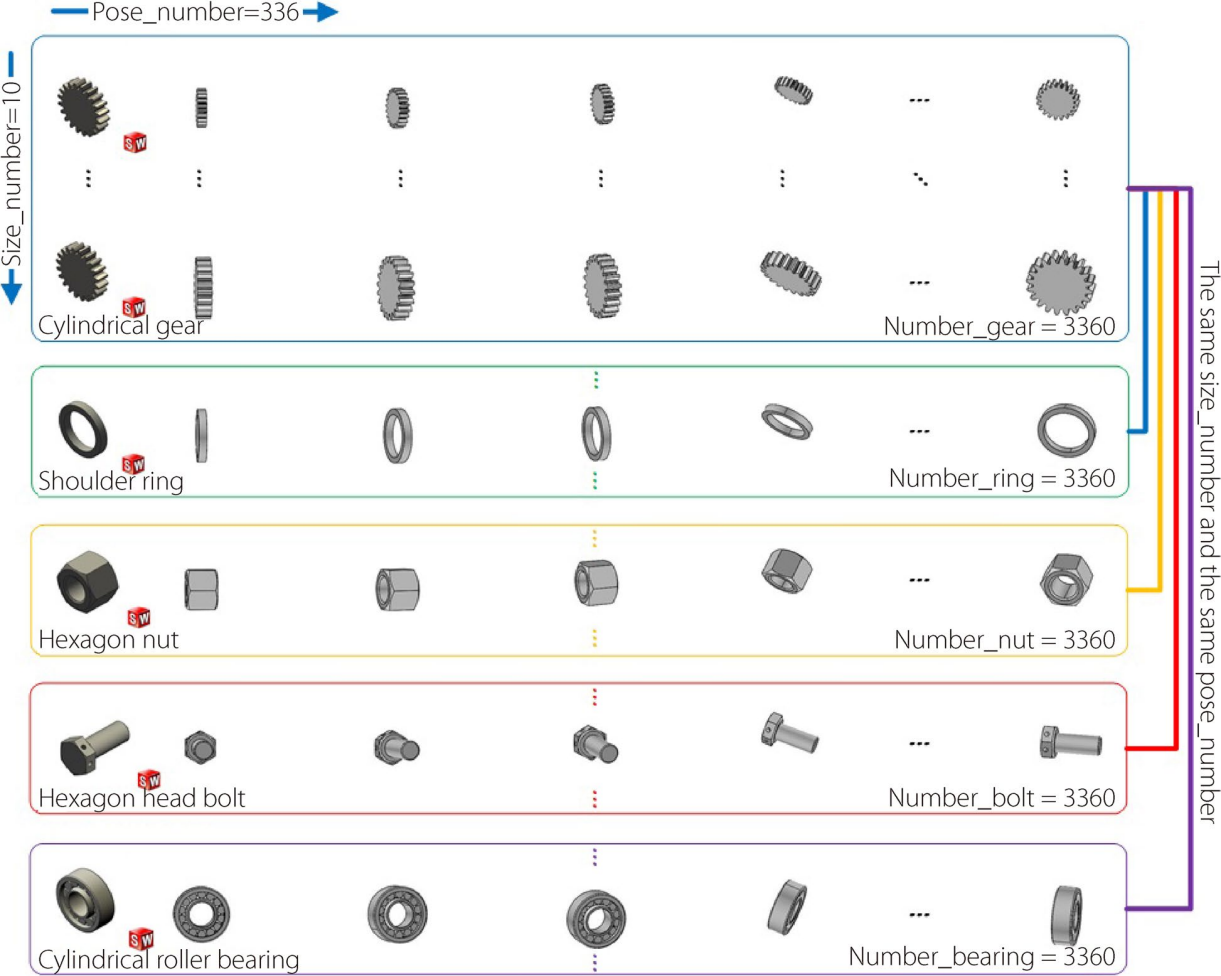
Standard dataset of five typical parts

Using the aforementioned parameters and a standard white background, a 2D dataset associated with five typical industrial parts for 3D reconstruction was constructed, as shown in Fig. 3. In this figure, the abscissa axis is sampled from 336 poses of every part, and the vertical axis is sampled from five typical parts, each with 10 sizes. Specifically, “One-Hot” labels are used in the sample image to complete the construction of the dataset. Finally, the dataset is divided into three parts: training, validation, and testing data with ratios of 81%, 9%, and 10%, respectively.

### NURBS-based shape reconstruction

This subsection discusses the reconstruction of a 3D shape from a single image and proposes a vector shape reconstruction approach for industrial parts. The proposed method mainly comprises three parts: First, a classification recognition network to distinguish the classes of industrial parts is designed; Second, CAD-ClassNet and CAD-ReconNet are proposed for industrial parts; Finally, based on CAD-ReconNet, the feature standard library of poses is derived, and the parameters of poses and sizes are obtained from feature analysis.

In conclusion, the proposed net comprises two parts: class and reconstruction prediction, where CAD-ClassNet is used to determine the type of the reconstructed part, and the model dimensions can be predicted using CAD-ReconNet.

#### CAD-ClassNet

**Fig. 4:**
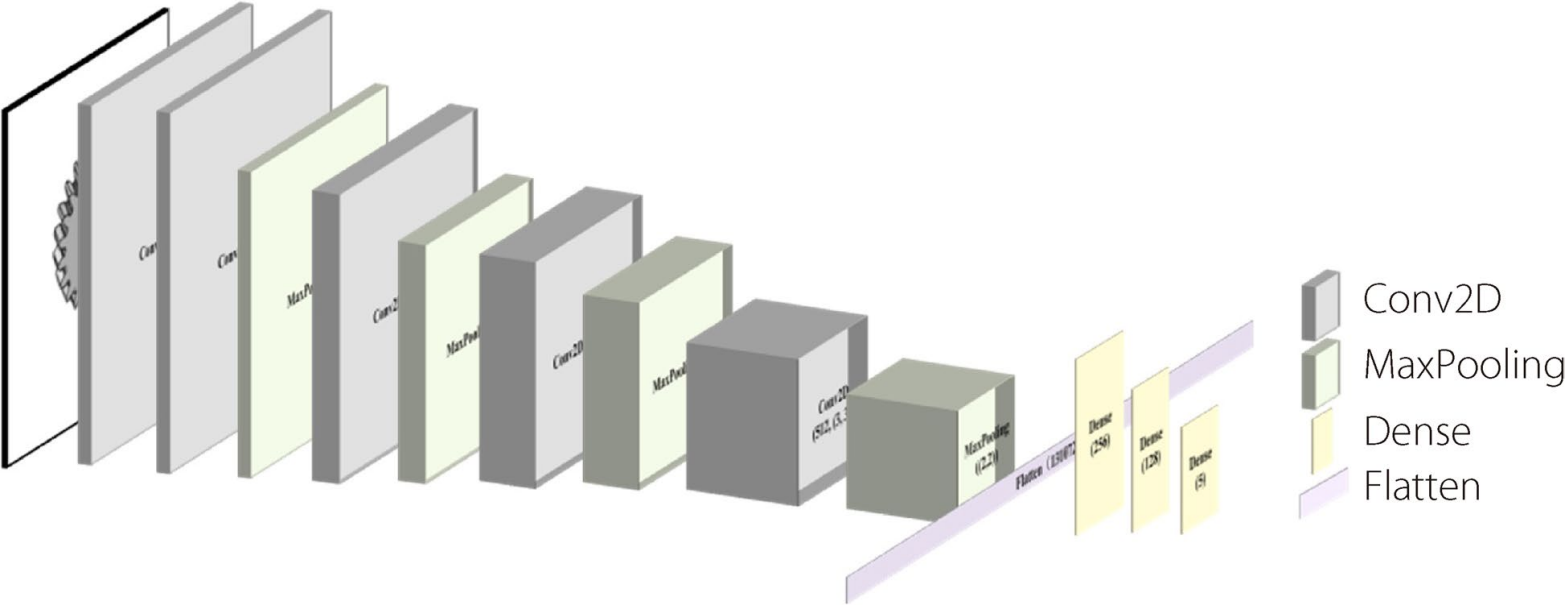
Structure of CAD-ClassNet

To determine the type of reconstructed part, a new network, CAD-ClassNet, was designed, whose structure is shown in Fig. 4. Five convolution layers and four maximum pooling layers were used to extract image features. The convolution kernel of the five convolution layers was 3 × 3 and the channel numbers were 64, 64, 128, 256, and 512, respectively. The pool size of the four pooling layers was 2 × 2. Thereafter, the 2D output to a one-dimensional vector was flattened and three dense layers were used to complete the classification of the industrial parts.

In CAD-ClassNet and CAD-ReconNet, the softmax activation function in the last layer and the ReLU function in the other convolution layers and full connection layers were used. In particular, the cross-entropy cost function shown in Eq. (2) was used as follows:

2 $$C = - \frac{1}{n}\sum\limits_{x}^ {\left[ {y\ln a + \left( {1 - y} \right)\ln \left( {1 - a} \right)} \right]}$$

where *x* denotes the input sample, *y* denotes the actual label, *a* denotes the predicted label, and *n* denotes the total number of samples.

#### Extraction of poses

**Fig. 5:**
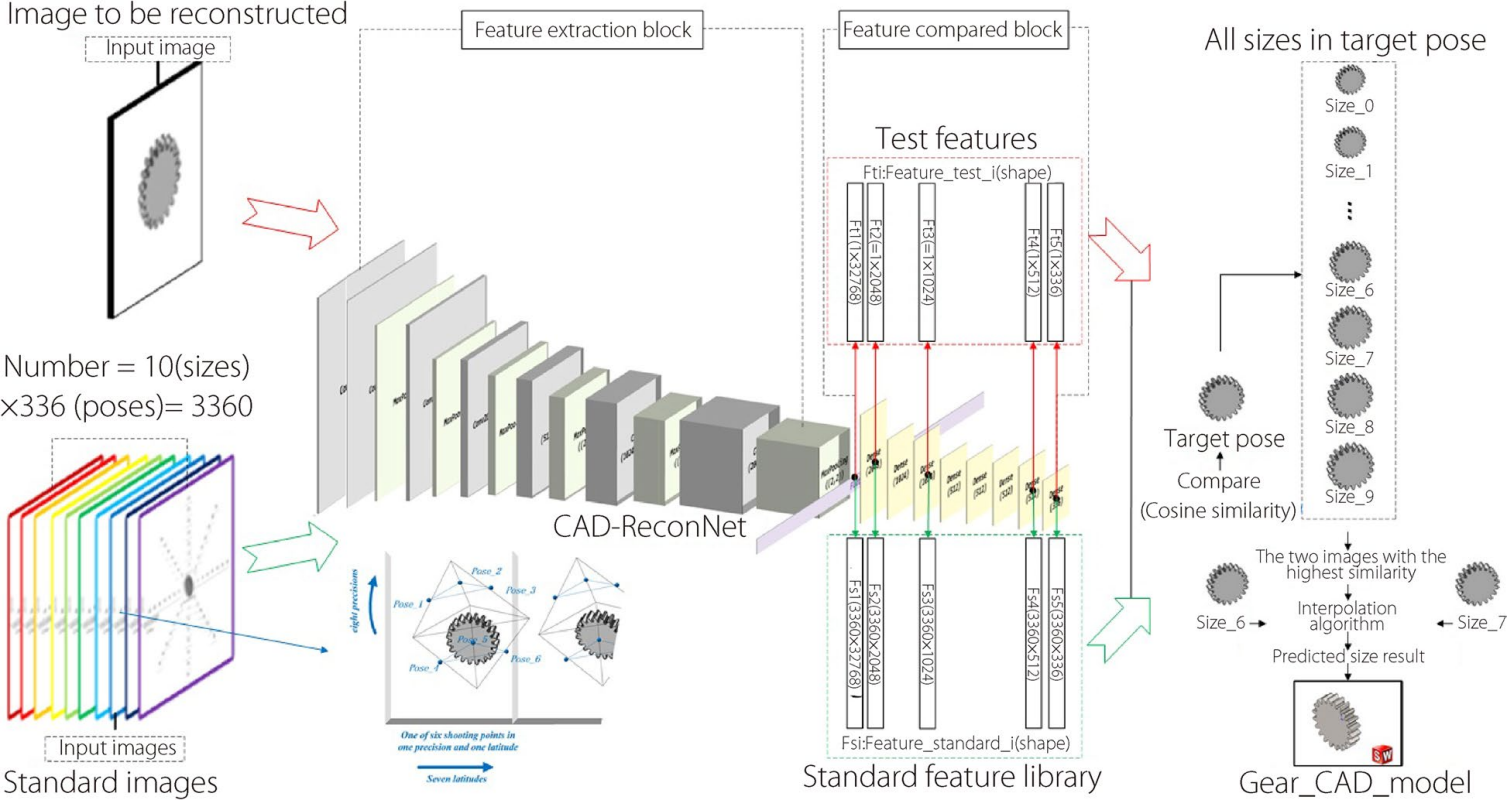
Extraction of parameters and reconstruction of gears

Gears are used as an example to represent the concept of shape reconstruction, as shown in Fig. 5. Each class of the five typical industrial parts in this study contains 3360 2D images. The net training and testing processes were completed using this dataset.

The pose determination of parts of 2D images can be regarded as a multiclassification problem. VGGNet and ResNet are often used as feature extractors and classifiers; however, these architectures did not work well on the dataset used in this study. A new network was constructed, CAD-ReconNet (Fig. 5), for pose recognition, in which there are several dense and batch normalization layers. In particular, the net is divided into two parts, a feature extraction block and a feature comparison block, which are described in detail as follows:

Feature extraction block: It comprises seven convolution layers with 3 × 3 convolution kernels and six maximum pooling layers with a stride of two. Among the seven convolutional layers, the channel number of the first two layers was 64, and that of the subsequent convolutional layers increased by a ratio of two. In this step, 2048 feature maps with a size of 4 × 4 were extracted.

Feature compared block: In the second step, the obtained 32,768 dimensional vectors were compressed into 2048, 1024, and 512 dimensional one through one, two, and four full connection layers, respectively. Finally, the input images were classified into 336 poses. In this process, the feature vectors in five different dimensional spaces (32,768, 2048, 1024, 512, and 336) were used as unique features for different poses, and they were also used for pose comparison among different images.

### NURBS-based reconstruction

Based on CAD-ReconNet, it is possible to extract the features of the standard dataset of industrial parts and thereafter construct the standard feature library used in pose prediction. The basic idea behind obtaining the dimensions is as follows: (1) Extract the pose feature of the reconstructed 2D image; (2) Compare the pose feature map extracted from the input image with the feature map in the standard feature library and determine the pose information contained in the 2D image; (3) Calculate the similarity between the input image and standard image in the same pose, and thereafter predict the dimensions of the parts according to the similarity. To evaluate the similarity between images, the cosine similarity strategy computed using Eq. (3) was used as follows:

3 $$\cos \left( \theta \right) = \frac{{\sum\limits_{i = 1}^{n} {\left( {A_{i} \times B_{i} } \right)} }}{{\sqrt {\sum\limits_{i = 1}^{n} {\left( {A_{i} } \right)^{2} } } \times \sqrt {\sum\limits_{i = 1}^{n} {\left( {B_{i} } \right)^{2} } } }}$$

where *A* and *B* denote the image feature vectors. The pose vector dimension is 336.

**Fig. 6:**
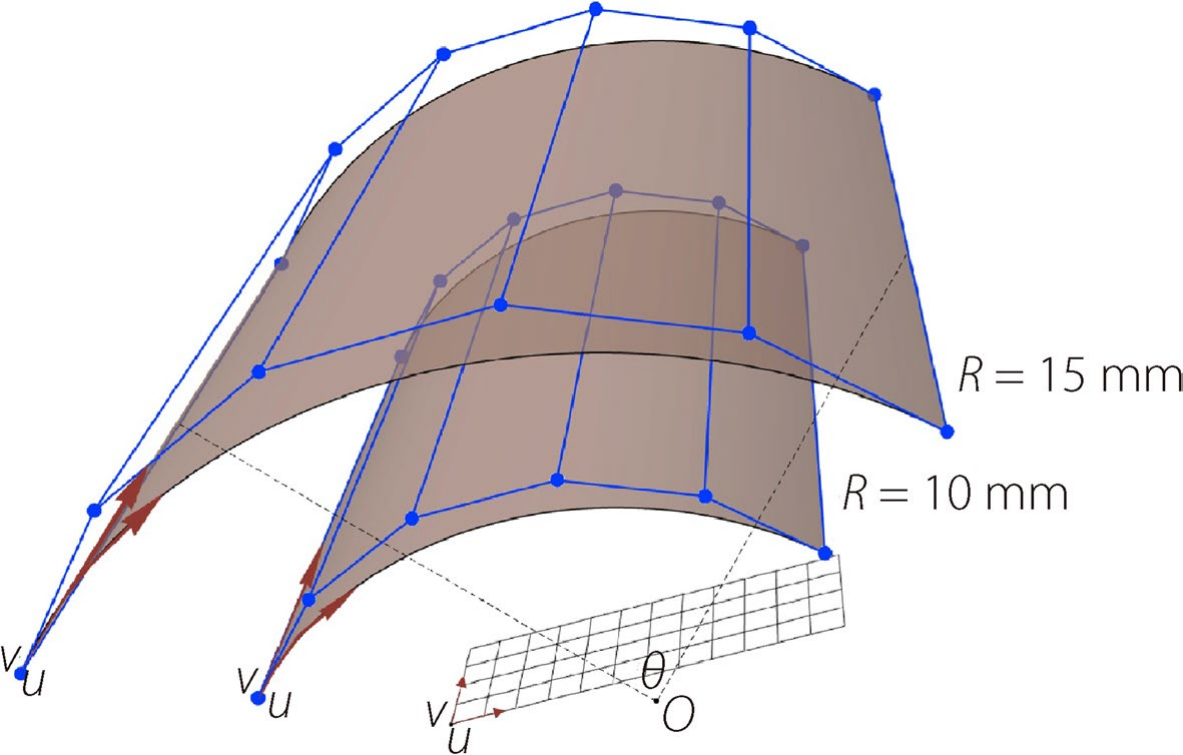
Scale transformation with same knot vector

Two poses from the images associated with the highest similarity were selected as the primary positions. Thereafter, a subset of standard images with the same position but of different sizes were obtained. By selecting the two sample images associated with the highest similarity, the part size that needed to be 3D reconstructed was predicted. The interpolation coefficients were calculated using Eq. (4), and the final predicted results for the part dimensions were obtained via linear interpolation.

4 $$\begin{array}{*{20}c} {Sim_{i} = \frac{{\left( {1 - \cos \left( {\theta_{{2{ - }i}} } \right)} \right)}}{{\sum\nolimits_{i} {\left( {1 - \cos \left( {\theta_{i} } \right)} \right)} }}} & {\left( {i = 1,2} \right)} \\ \end{array}$$

where $\cos \left( {\theta_{i} } \right)$ denotes the cosine similarity between images. Furthermore, if the part type is determined, its corresponding control points can be obtained by scale transformation based on the dimension (Fig. 6) and standard model of typical parts. The 3D reconstruction model of the part can be defined using the control points and the same knot vector.

## Results and Discussion

### Class prediction

To improve the accuracy of class recognition, random rotation, clipping, and bright transformation were applied to the part images for data enhancement. The proposed network has strong convergence and classification ability and achieves good generalization results. Through the testing dataset, the results demonstrate that the classification accuracy of industrial parts is 100%.

### Pose prediction

**Fig. 7:**
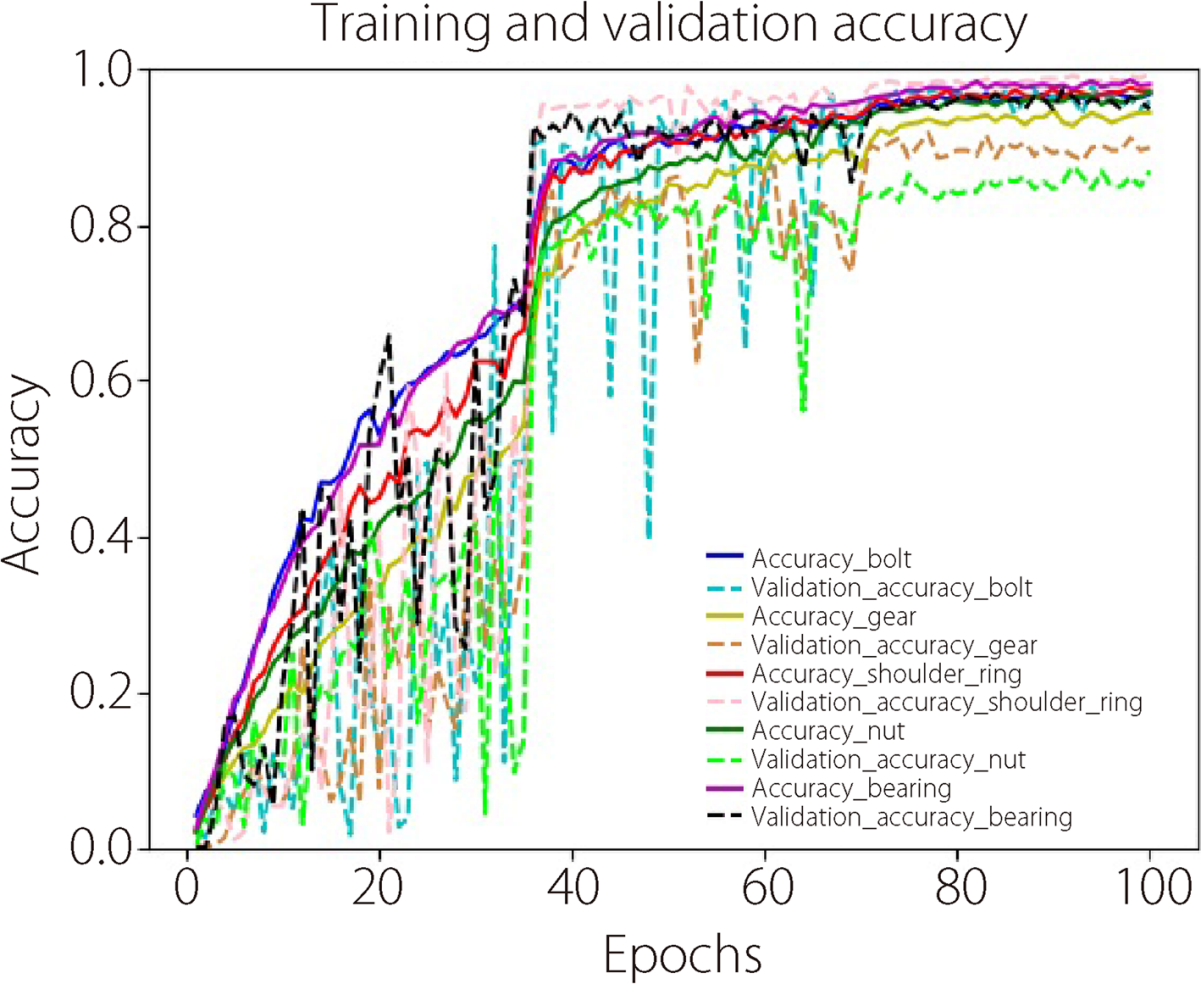
Accuracy of CAD-ReconNet

Each part class has its own standard dataset. In the dataset, there are 336 different poses, and each pose has 10 different sizes. In other words, there were several classifications in the dataset, but the number of samples in each classification was relatively small. Batch normalization was used to accelerate the convergence. Strategies for learning the rate gradient decline were used, and the initial value was set to 0.001. A total of 100 epochs were iterated, and the learning rate was changed to 35 and 70 epochs at a ratio of 0.1. The results obtained for this net structure and parameters are shown in Fig. 7.

It is possible to infer that the net loss and accuracy first have large fluctuations because the initial learning rate is set to a larger value (Fig. 7). As the learning rate decreased, the net fluctuation decreased and the net tended to converge. During training and testing, the accuracy and loss of information for each part were monitored (Table 2).

**Table 2:** Performance of CAD-ReconNet training and testing

Parameters	Minimum training loss	Maximum training accuracy	Minimum verification loss	Maximum verification accuracy	Maximum testing accuracy
Hexagon head bolt	0.1146	96.91%	0.0704	97.69%	97.92%
Cylindrical gear	0.2161	94.60%	0.2239	91.75%	89.29%
Shoulder ring	0.1164	97.50%	0.0444	99.34%	99.70%
Hexagon nut	0.1373	96.95%	0.3928	87.46%	81.85%
Cylindrical roller bearing	0.0782	98.49%	0.0968	97.36%	95.24%

The maximum testing accuracy of the hexagonal nut is slightly higher than 80%, whereas those of the other parts are close to or higher than 90% (Table 2). The strong structural symmetry and small size differences in each direction may explain the low reconstruction accuracy of the hexagonal nut. Overall, it is possible to infer that CAD-ReconNet achieves good results and generalization performance.

CAD-ReconNet was compared with ResNet-18 (batch size = 20) and ResNet-34 (batch size = 10). Table 3 summarizes the results, and it can be observed that the proposed network converged better. In other words, the features extracted using the proposed network were highly reliable.

**Table 3:** Comparison of CAD-ReconNet and other networks

Network	Parameters	Maximum training accuracy	Maximum verification accuracy	Maximum testing accuracy
CAD-ReconNet	Hexagon head bolt	96.91%	97.69%	97.92%
	Cylindrical gear	94.60%	91.75%	89.29%
	Shoulder ring	97.50%	99.34%	99.70%
	Hexagon nut	96.95%	87.46%	81.85%
	Cylindrical roller bearing	98.49%	97.36%	95.24%
ResNet-18	Hexagon head bolt	94.56%	94.72%	92.56%
	Cylindrical gear	91.25%	77.89%	84.52%
	Shoulder ring	85.63%	84.16%	85.12%
	Hexagon nut	86.40%	73.93%	78.27%
	Cylindrical roller bearing	95.11%	91.09%	87.50%
ResNet-34	Hexagon head bolt	98.31%	96.70%	95.54%
	Cylindrical gear	92.65%	83.83%	82.44%
	Shoulder ring	98.57%	92.74%	92.86%
	Hexagon nut	93.01%	83.83%	82.14%
	Cylindrical roller bearing	95.33%	93.07%	87.20%

### Dimension analysis

For industrial parts, the key problem in 3D reconstruction is obtaining the model dimensions, and its accuracy directly affects the validity of the final shape results. Therefore, the accuracy of the size prediction is at the core of net training and testing. For each part, two test sizes (Table 1) were selected and a test set (672 images) was constructed to verify the effectiveness of the proposed method. The proposed approach achieves a better result in size prediction for every typical part (Table 4).

**Table 4:** Accuracy of the 3D reconstruction (percentages of every relative error interval)

Relative error industrial parts	0%–0.05%	0.05%–0.1%	0.1%–0.5%	0.5%–1.0%	> 1.0%
Hexagon head bolt	14.73	12.50	51.04	8.78	12.95
Cylindrical gear	42.26	25.74	18.30	1.19	12.50
Shoulder ring	30.06	14.29	52.64	0.15	3.87
Hexagon nut	1.19	2.23	73.36	2.23	20.37
Cylindrical roller bearing	8.78	26.34	46.58	9.38	8.93

**Fig. 8:**
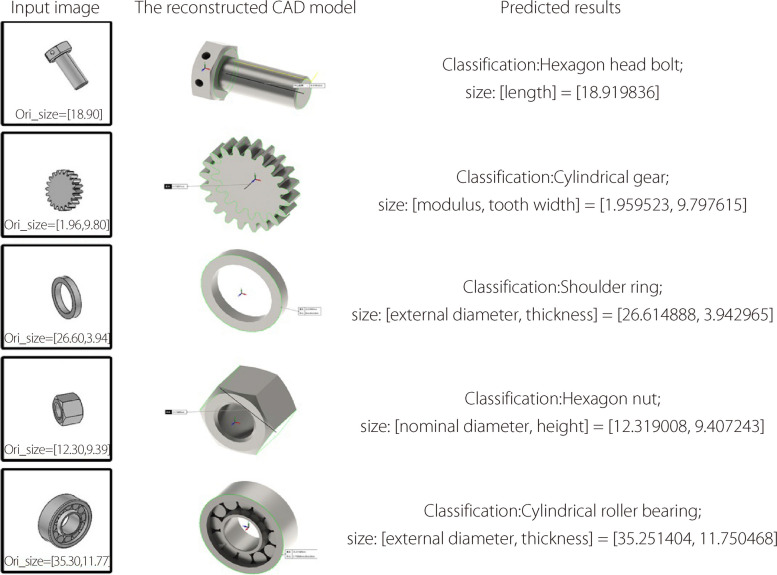
Parameter prediction and 3D reconstruction

**Fig. 9:**
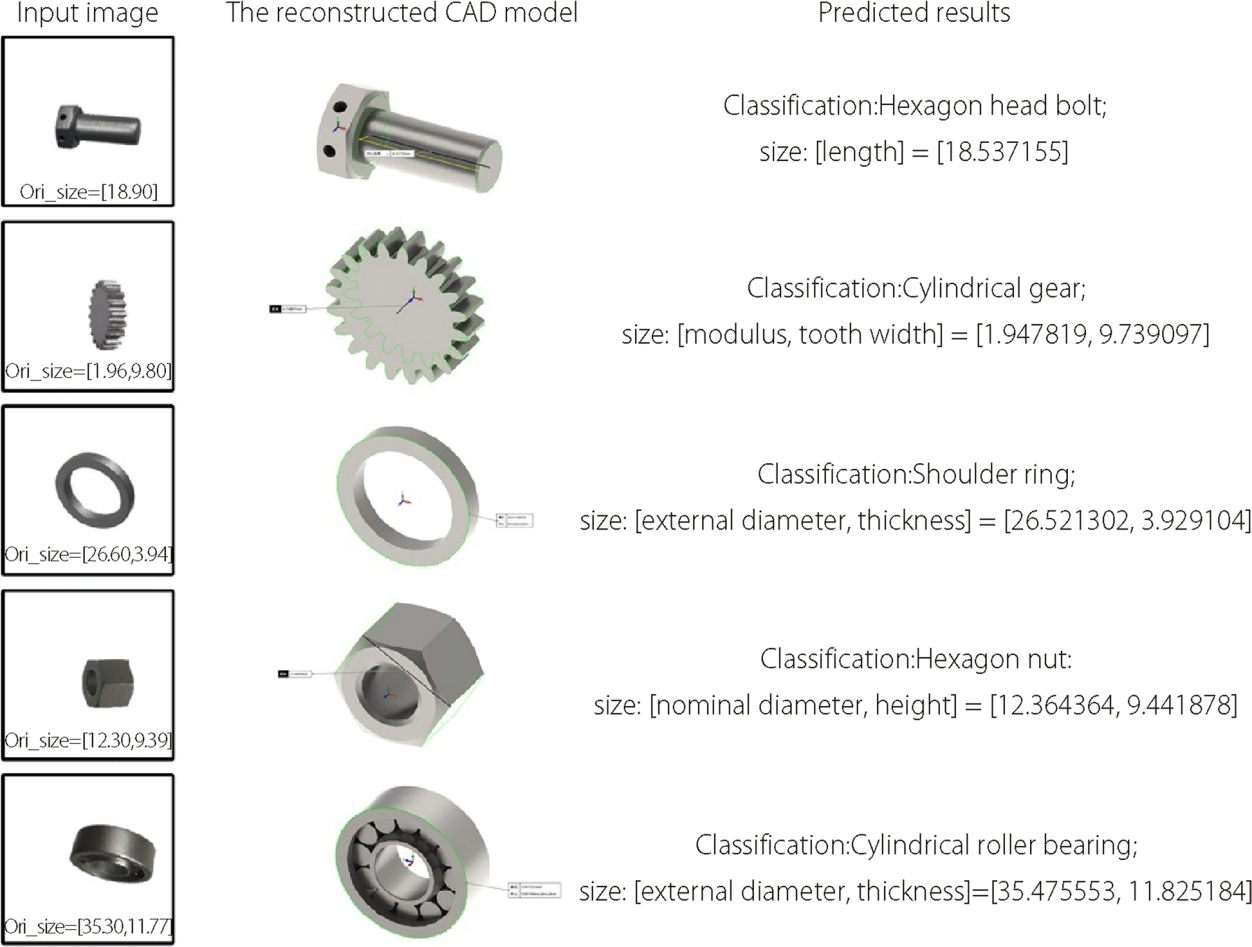
3D reconstruction of real parts

From the predicted reconstruction parameters of the typical parts, it is possible to complete the 3D reconstruction based on NURBS from a single image (Fig. 8). To further verify the feasibility of the vector shape reconstruction method, testing images captured from real industrial parts and reconstructed 3D models with high accuracy were used (Fig. 9). Before testing, images from the cameras in the natural scene were preprocessed using background culling and regularization.

**Fig. 10:**
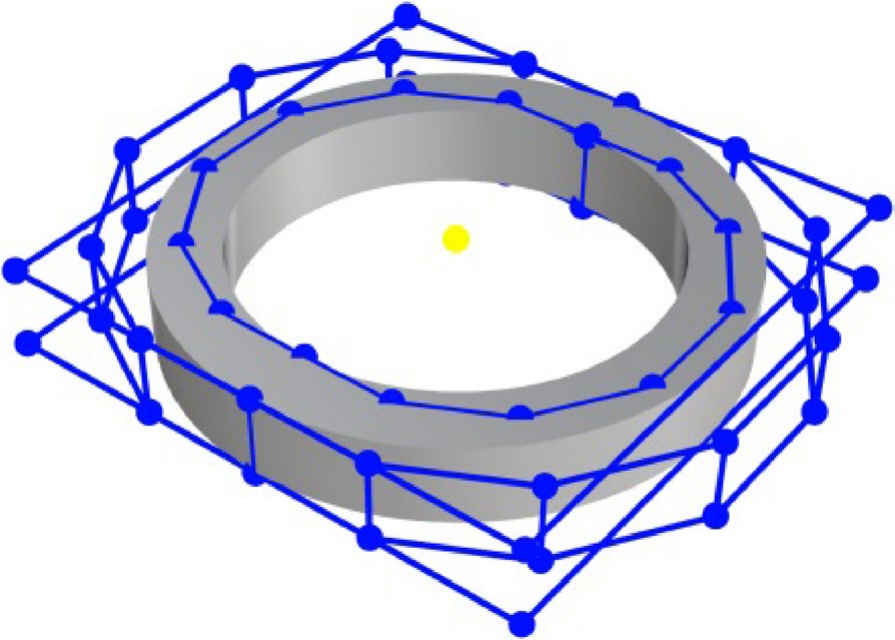
NURBS-based geometric modeling of a shoulder ring

According to the results in Figs. 8 and 9, the accuracy of shape reconstruction is less than 0.1 mm from a single image. The proposed system takes an average of 19–20 s to complete the reconstruction of an image with a “NVIDIA GeForce RTX 3070” GPU, an “Intel Core i5-10400F @ 2.90 GHz” CPU, and “16 GB” memory size. Additionally, it outputs the NURBS control points required to define a mechanical part. Thereafter, 3D reconstruction was achieved using geometric modeling based on NURBS. Figure 10 shows the control points and CAD model of the shoulder ring. From these experiments, the proposed approach can reconstruct industrial parts with high accuracy and efficiency using a single image. Each type of part has known control point structures. Given a model, it is possible to only scale the control edges to fit them to the real dimensions of the part.

## Conclusions

In this study, a 3D reconstruction system for industrial parts based on NURBS was constructed, which can achieve the intelligent computation of parameters. Using the predicted parameters, it is possible to reconstruct the corresponding 3D shapes of the industrial parts, which achieves vector reconstruction from a single image. The main contributions of this study are as follows: first, a dataset of 2D images for typical industrial parts is constructed, including hexagon head bolts, cylindrical gears, shoulder rings, hexagon nuts, and cylindrical roller bearings; second, a deep learning algorithm for the parameter extraction of 3D industrial parts is developed using two new nets: CAD-ClassNet and CAD-ReconNet; finally, the 3D shape reconstruction of parts based on NURBS is presented. Examples were provided to illustrate the accuracy and efficiency of the proposed reconstruction approach.

## Data Availability

Not applicable.

## Abbreviations

3D: Three-dimensional
2D: Two-dimensional
CAD: Computer aided design
NURBS: Non-uniform rational B-splines
